# MiR-154-5p-MCP1 Axis Regulates Allergic Inflammation by Mediating Cellular Interactions

**DOI:** 10.3389/fimmu.2021.663726

**Published:** 2021-05-31

**Authors:** Misun Kim, Hyein Jo, Yoojung Kwon, Myeong Seon Jeong, Hyun Suk Jung, Youngmi Kim, Dooil Jeoung

**Affiliations:** ^1^ Department of Biochemistry, Kangwon National University, Chuncheon, South Korea; ^2^ Chuncheon Center, Korea Basic Science Institute (KBSI), Chuncheon, South Korea; ^3^ Institute of New Frontier Research, College of Medicine, Hallym University, Chuncheon, South Korea

**Keywords:** miR-154-5p, MCP1, exosomes, cellular interactions, allergic inflammation

## Abstract

In a previous study, we have demonstrated that p62, a selective receptor of autophagy, can regulate allergic inflammation. In the present study, microRNA array analysis showed that miR-154-5p was increased by antigen (DNP-HSA) in a p62-dependent manner in rat basophilic leukemia cells (RBL2H3). NF-kB directly increased the expression of miR-154-5p. miR-154-5p mediated *in vivo* allergic reactions, including passive cutaneous anaphylaxis and passive systemic anaphylaxis. Cytokine array analysis showed that antigen stimulation increased the expression of MCP1 in RBL2H3 cells in an miR-154-5p-dependent manner. Reactive oxygen species (ROS)-ERK-NF-kB signaling increased the expression of MCP1 in antigen-stimulated RBL2H3 cells. Recombinant MCP1 protein induced molecular features of allergic reactions both *in vitro* and *in vivo*. Anaphylaxis-promoted tumorigenic potential has been known to be accompanied by cellular interactions involving mast cells, and macrophages, and cancer cells. Our experiments employing culture medium, co-cultures, and recombinant MCP1 protein showed that miR-154 and MCP1 mediated these cellular interactions. MiR-154-5p and MCP1 were found to be present in exosomes of RBL2H3 cells. Exosomes from PSA-activated BALB/C mouse induced molecular features of passive cutaneous anaphylaxis in an miR-154-5p-dependent manner. Exosomes from antigen-stimulated RBL2H3 cells enhanced both tumorigenic and metastatic potentials of B16F1 melanoma cells in an miR-154-5p-dependent manner. Exosomes regulated both ROS level and ROS mediated cellular interactions during allergic inflammation. Our results indicate that the miR-154-5p-MCP1 axis might serve as a valuable target for the development of anti-allergy therapeutics.

## Introduction

Bioactive mediators released by mast cells can mediate allergic inflammation (1). Anaphylaxis involves decreased rectal temperatures, increased vascular permeability, and β-hexosaminidase activity (2). Mast cells and basophils can mediate IgE-dependent anaphylaxis (3). Cellular interactions involving mast cells and many other immune cells can also mediate anaphylaxis (4, 5). These reports suggest that soluble factors, including exosomes and cytokines may promote anaphylaxis by mediating these cellular interactions.

Autophagy is necessary for the degranulation of mast cells (6, 7) and pathogenesis of allergic inflammation such as asthma (8). B cell autophagy contributes to the pathogenesis of asthma (9). Autophagy plays a critical role in asthma (10). Allergen-induced ROS signaling is necessary for autophagy in asthma (8). Allergic inflammation such as anaphylaxis is closely associated with enhanced autophagy (4). Thus, roles of autophagy-related genes in anaphylaxis merit further investigation.

Previous work has shown that exosomes can mediate cellular interactions during allergic inflammation (4). Eosinophil-derived exosomes contribute to the pathogenesis of asthma (11). Mesenchymal stem cell exosomes are known to exert a negative effect on allergic asthma (12). P62, a receptor of autophagy, can mediate allergic reactions. It is present in the exosomes (4). Secretion of exosomes can lead to enhanced autophagy (13). These reports suggest that exosomal molecules may regulate allergic inflammation by mediating cellular interactions.

MicroRNAs (miRNAs) play a critical role in allergic inflammations (14). Downregulation of Dicer, a key enzyme in miRNA biogenesis, can inhibit degranulation. It is known that miRNAs can regulate mast cell degranulation (15). miR-21-5p is necessary for allergic airway remodeling (16). miR-126 can promote mast cell proliferation (17). Exosomal miRNAs from the airway epithelium contribute to the development of a Th2 response in asthma (18). miR-122 can inhibit cellular interactions during allergic inflammation by decreasing the expression of SOCS1 (2). miR-135-5p can inhibit allergic inflammation by regulating cellular interactions (4). These reports suggest that exosomal miRNAs may regulate anaphylaxis by mediating cellular interactions.

In this study, we identified miR-154-5p as a downstream target of p62. MiR-154-5p was necessary for the increased expression of MCP1 in anaphylaxis. The miR-154-5p-MCP1 axis was necessary for tumorigenic and metastatic potentials of cancer cells enhanced by anaphylaxis. MiR-154-5p and MCP1 were found to be localized in the exosomes. The miR-154-5p-MCP1 axis was also necessary for cellular interactions mediated by exosomes during allergic inflammation and tumorigenic potential of cancer cells enhanced by anaphylaxis. Our data suggest the miR-154-5p-MCP1 axis can be employed for development of anti-allergy therapeutics.

## Materials and Methods

### Materials

Oligonucleotides were purchased from Bioneer Company (South Korea). We purchased chemicals, DNP-HSA and DNP-specific IgE antibody from Sigma. We purchased anti-mouse and anti-rabbit IgG-horseradish peroxidase-conjugated antibody from Pierce. We purchased all other antibodies from Cell Signaling Company (Beverly, MA). We purchased Lipofectamine and Plus™ reagent from Invitrogen. We purchased recombinant MCP1 protein from R&D system.

### Cell Culture

We purchased rat basophilic leukemia (RBL2H3) cells from the Korea Cell Line Bank (Seoul, Korea). RBL2H3 cells and B16F1 Cells were grown as described (19). Cells were maintained at 37°C in 5% CO2. Isolation of lung mast cells and lung macrophages was performed according to standard procedures (19).

### Mice

We purchased five-week-old female BALB/C mice from Nara Biotech (Seoul, Korea). Mice were maintained in specific pathogen-free conditions. All animal experiments were approved by the Institutional Animal Care and Use Committee (IACUC) of Kangwon National University.

### Transfection

For miR-154-5p inhibition, 10 nM oligonucleotide (inhibitor) along with jetPRIME^®^ (Polyplus, cat.114-15) was transfected into cells. The sequences used were 5ʹ-UAGGUUAUCCGUGUUGCCUUCG-3ʹ (miR-154-5p inhibitor) and 5′-TAACACGTCTATACGCCCA-3′ (control inhibitor). The sequences of siP62 are [5′- GACGAUGACUGGACACAUU -3′ (sense) and 5′- AAUGUGUCCAGUCAUCGUC -3′ (antisense)]. The negative control siRNA was purchased from Bioneer Company (cat.SN-1002). For *in vivo* transfections, *in vivo*-jetPEI^®^ (Polyplus, cat.201-10G) was used.

### β-Hexosaminidase Activity Assays

The β-hexosaminidase activity assay was performed according to standard procedures with some modifications (20). The detailed procedures are described in supplemental methods.

### The Levels of PGE2, Histamine Release, and MCP1

The levels of PGE2 and the amount of histamine released were measured using ELISA kit (Abcam, UK). Reaction product was measured colorimetrically with a microplate reader. MCP1 level was determined employing ELISA kit (Abcam).

### Immunoblot and Immunoprecipitation

Immunoblot and immunoprecipitation were performed as described with some modifications (21, 22). The detailed procedures are described in supplemental methods. The following primary antibodies were used: Lyn (sc-15, Santa Cruz), NF-κB (8242S, Cell Signaling), IκB (4814S, Cell Signaling), p IκB^ser32^ (2859S, Cell Signaling), FcϵRIβ (sc-398863, Santa Cruz), SOCS1 (ab9870, Abcam), Beclin1 (sc-48341, Santa Cruz), pBeclin1^Ser15^ (84966S, Cell Signaling), LC3 (12741S, Cell Signaling), HDAC3 (3949S, Cell Signaling), MCP-1 (ab25124, Abcam), CD163 (ab182422, Abcam), iNOS (13120S, Cell Signaling), CCR2 (NBP2-67700, NOVUSBIO), ERK1/2 (67170-1, Proteintech), pERK1/2^Tyr204^ (sc-7383, Santa Cruz), E-Cadherin (3195S, Cell Signaling), Vimentin (5741S, Cell Signaling), Alix (2171S, Cell Signaling), Beclin1 (sc-48341, Santa Cruz), IgG (sc-2025, Santa Cruz), Snail (sc-271977, Santa Cruz), CD63 (sc-5275, Santa Cruz), TSG101 (sc-7964, Santa Cruz), FAK (sc-558, Santa Cruz), and CD81 (sc-166029, Santa Cruz); Actin (A2228, Sigma), p62 (ab56416, Abcam).

The following secondary antibodies were used: Anti-mouse HRP secondary antibody (31430, Invitrogen), anti-goat HRP secondary antibody (31402, Invitrogen), anti-rabbit HRP secondary antibody (ADI-SAB-300-J, Enzo), anti-rabbit Alexa Fluor 488 secondary antibody (A11008, Invitrogen), anti-rabbit Alexa Fluor 546 secondary antibody (A11035, Invitrogen).

### miRNA Array

The miRNA Array III (Signosis, CA, United States) was used for miRNA expression analysis. Total miRNA was hybridized to 132 miRNA oligonucleotide probes. The level of miRNA was determined by Streptavidin-HRP chemiluminescence.

### Cytokine Array

Cytokine array analysis (Proteom Profiler™ Mouse Cytokine Array Kit) was performed as described (R&D system).

### Immunofluorescence Staining

Cells were fixed with paraformaldehyde for 10 min before being permeabilized in 0.4% Triton X-100 for 10 min. After blocking by 5% BSA, cells were incubated with anti-CD163 antibody (1:100; Abcam) or iNOS antibody (1:100; Santa Cruz Biotechnology) for 2 h and then incubated with anti-rabbit Alexa Fluor 488 (for detection of iNOS) or anti-goat Alexa Fluor 546 (for detection of CD163) secondary antibody for 1 h. Fluorescence images were observed and captured using a confocal laser scanning microscope and software (Fluoview version 2.0) with a X 60 objective (Olympus FV300, Tokyo, Japan).

### Chromatin Immunoprecipitation (ChIP) Assay

Cell lysates were incubated with NF-κB antibody and NF-κB-target gene complex was purified by protein G beads. PCR was done with specific primers of the miR-154-5p promoter-1 [5′-CACAAGGGTCTTCCTTTCCTTC -3′ (sense) and 5′-CAGGCAGCAAGCAGACTATT -3′ (antisense)], miR-154-5p promoter-2 [5′-GCTTTTGAACACTGGGGACTC -3′ (sense) and 5′-﻿AGCTGGCATTGCAATTAGGC-3′ (antisense)], and miR-154-5p promoter-3 [5′-AAGGTACCCTGAACGTTTGC -3′ (sense) and 5′-﻿CTAAGGGTCCTTACGGGGTC -3′ (antisense)] sequences were used.

### miRNA Extraction and Quantitative Real-Time PCR

Total miRNA was isolated with the miRNeasy Micro Kit (Qiagen, CA, United States). The extracted miRNA was reverse transcribed using a miScript II RT Kit (Qiagen, CA, United States) with universal RT primer. The expression level of miR-154-5p was quantified with SYBR Green Master Mix (Qiagen, CA, United States) using a miRNA-specific forward primer and universal reverse primer. The relative miRNA expression level was calculated using the comparative 2^-ΔΔCT^ method (ΔCT = CT_miR_ -CT_reference_). The sequences of miR-154-5p forward primer are 5′TAGGTTATCCGTGTTGCCTTCG-3′.

### Chemo Invasion and Migration Assays

Transwell chamber system with 8-μm pore polycarbonate filter inserts (CoSTAR, Acton, MA) was used for determination of invasive potential. The lower and upper sides of the filter were coated with gelatin and matrigel, respectively. Trypsinized cells (5 × 10^3^) in the serum-free RPMI 1640 medium containing 0.1% bovine serum albumin were added to each upper chamber of the transwell. RPMI 1640 medium containing 10% fetal bovine serum was placed in the lower chamber and cells were incubated at 37°C for 16 h. For determination of migration potential, the lower sides of the filters were coated with gelatin.

### Passive Cutaneous Anaphylaxis

BALB/C mice were intradermally injected with DNP-specific IgE (0.5 μg/kg). Twenty-four hours later, mice were intravenously injected with DNP-HSA (250 μg/kg) and 2% (v/v) evans blue solution. One hour after injection with evans blue solution, evans blue dye was extracted from each dissected ear in 700 μl of acetone/water (7:3) overnight. The amount of evans blue in the extracts was determined colorimetrically at 620 nm. To examine the effect of miR-154-5p, BALB/C mice were given an intravenous injection of miR-154-5p inhibitor (3.5 μg/kg) and an intradermal injection of DNP-specific IgE (0.5 μg/kg).

### Passive Systemic Anaphylaxis

Passive systemic anaphylaxis was induced according to the standard procedures (2). To determine effect of miR-154-5p on, BALB/C mice were intravenously injected with DNP-specific IgE (0.5 μg/kg) along with miR-154-5p inhibitor or control inhibitor (3.5 μg/kg). The next day, BALB/C mice were intravenously injected with PBS or DNP-HSA (250 μg/kg). Rectal temperatures were measured using a digital thermometer.

### Isolation and Characterization of Exosomes

Exosomes were purified using Exoquick-TC reagent (System Biosciences, Mountain View, CA). Tecnai T10 transmission electron microscope (FEI, USA) was employed for observation of exosomes. Zetasizer (Malvern Corp., UK) was employed for measuring the size of exosomes.

### Labeling and Internalization of Exosomes

PKH67 Fluorescent Cell Linker kits (Sigma-Aldrich, St. Louis, MO) were used for labeling of exosomes. RBL2H3 cells were plated out onto coverslip (2 × 10^4^ cells). The next day, coverslips were washed in PBS, and each medium containing PKH67-labeled exosomes or PKH67-unlabeled exosomes was added into RBL2H3 cells for 24 h. The coverslips were then washed three times in PBS, and fixing solution (4% paraformaldehyde) then added to the coverslips for 15 min. The coverslips were then washed three times in PBS. A confocal laser scanning microscope LX70 FV300 05-LPG-193 (Olympus) was used for visualization of cells.

### The Presence of MCP1 in the Exosomes of Antigen-Stimulated RBL2H3 Cells

Immuno-EM was performed to examine the presence of MCP1 in the exosomes. Primary rabbit or/and mouse antibodies (Anti-MCP1 or/and Anti-TSG1 antibodies) at 1:20 dilutions were used. The grid was incubated in secondary antibodies, anti- Rabbit IgG conjugated to 10 nm and anti-mouse IgG conjugated to 25 nm (AURION, Holland) diluted 1:20 in 0.1% BSA-PBS. The sample grids were stained with uranyl acetate and lead citrate. The sectioned and immune-gold labeled grids were examined using a Tecnai T10 transmission electron microscope (FEI, USA) operated at 100 kV and JEOL-2100F transmission electron microscope (JEOL, USA) operated at 200 KV.

### Reactive Oxygen Species (ROS) Measurement

DCF-DA solution (10 μM) was added to each well. DCF-DA was added 30 minutes after addition of DNP-HSA. The fluorescence of the 2’, 7’-dichlorofluorescein (DCF) product was detected and quantified by fluorescence microscope.

### Statistical Analysis

Data were analyzed using the GraphPad Prism statistics program (Version 7, GraphPad Prism software). Results are presented as means ± SE. Student’s t tests were performed for comparisons between two groups. One-way ANOVA was carried out for comparisons among three or more groups and was followed by Tukey’s *post hoc* test. Values were considered to be significant at p value less than 0.05.

## Results

### MiR-154-5p Mediates Allergic Inflammation *In Vitro*

**Figure 1:**
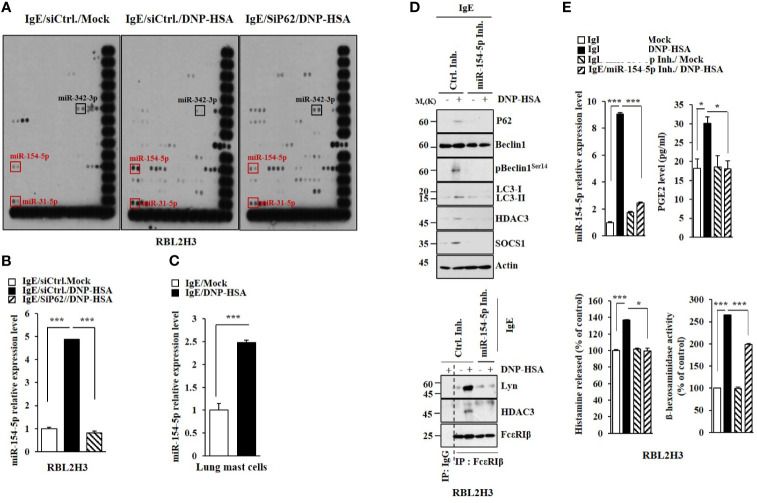
MiR-154-5p is necessary for *in vitro* allergic reactions. **(A)** RBL2H3 cells were transfected with indicated siRNA (each at 10 nM). The next day, cells were then sensitized with DNP-specific IgE (100 ng/ml) for 24 h followed by stimulation with DNP-HSA (100 ng/ml) for 1 h. miRNA array analysis was performed. The siCtrl. denotes control (negative) siRNA. **(B)** QRT-PCR analysis was performed. ***p<0.001. Average values of three independent experiments are shown. **(C)** IgE-sensitized lung mast cells were treated with DNP-HSA for 1 h. QRT-PCR was performed. ***p<0.001. Average values of three independent experiments are shown. **(D)** RBL2H3 cells were transfected with the indicated inhibitor (each at 10 nM). The next day, cells were sensitized with DNP-specific IgE for 24 h followed by stimulation with DNP-HSA for 1 h. Immunoprecipitation with isotype-matched IgG was also performed. Representative blots of three independent experiments are shown. **(E)** Levels of PGE2 and the amount of histamine released into growth medium were determined. ß-hexosaminidase activity assays and qRT-PCR were also performed. *p<0.05; ***p<0.001. Average values of three independent experiments are shown.

We have previously reported that p62, a selective receptor of autophagy, is necessary for allergic inflammation including anaphylaxis (4). miRNA array analysis was performed to identify targets of p62. It was found that p62 could regulate the level of miR-154-5p in antigen (DNP-HSA)-stimulated rat basophilic leukemia (RBL2H3) cells (**Figure 1A**). P62 might act as a negative regulator of miR-342-3p (**Figure 1A**). The effect of p62 on miR-154-5p expression was confirmed by qRT-PCR (**Figure 1B**). MiR-154-5p expression was also increased in antigen-simulated lung mast cells (**Figure 1C**). MiR-154-5p inhibitor prevented antigen from increasing allergic inflammation hallmarks (**Figure 1D**). An miR-154-5p inhibitor also prevented antigen from inducing interactions between FcϵRI and HDAC3 or Lyn (**Figure 1D**). The miR-154-5p inhibitor also exerted negative effects on the increase of prostaglandin E2 (PGE2) level, amount of histamine released, and ß-hexosaminidase activity in antigen-stimulated RBL2H3 cells (**Figure 1E**). Promoter sequences of miR-154-5p contain potential binding site for NF-kB (**Figure S1A**). BAY 11-7085, an inhibitor of NF-kB, exerted a negative effect on the expression of HDAC3 increased by antigen (**Figure S1B**). BAY 11-7085 also prevented antigen from increasing the expression of miR-154-5p (**Figure S1C**). NF-kB showed binding to the promoter sequences of miR-154-5p (**Figure S1D**). Thus, miR-154-5p can mediate allergic reactions *in vitro*.

### MiR-154-5p Mediates Anaphylaxis

**Figure 2:**
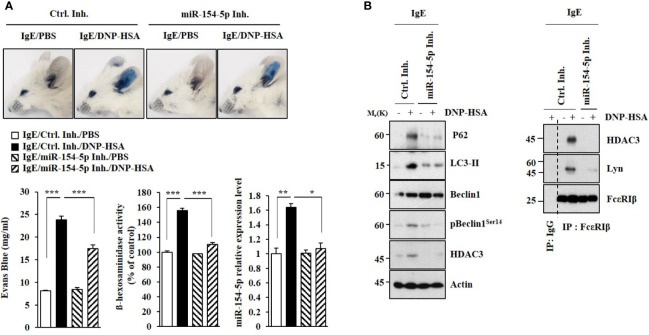
MiR-154-5p mediates PCA. **(A)** BALB/C mice were intradermally injected with DNP-specific IgE antibody (0.5 μg/kg) along with the indicated inhibitor (3.5 μg/kg). The next day, BALB/C mice were intravenously injected with PBS or DNP-HSA (250 μg/kg) along with 2% (v/v) Evans blue solution. The β-hexosaminidase activity assay and qRT-PCR analysis employing ear tissue lysates were performed. *p<0.05; **p<0.01; ***p<0.001. **(B)** Immunoblot and immunoprecipitation employing ear tissue lysates were performed. Representative blots of three independent experiments are shown.

**Figure 3:**
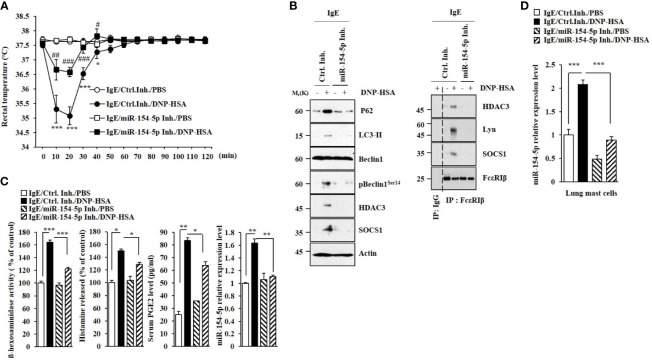
MiR-154-5p mediates PSA. **(A)** BALB/C mice were intravenously injected with DNP-specific IgE along with the indicated inhibitor (3.5 μg/kg). The following day, BALB/C mice were intravenously injected with DNP-HSA (250 μg/kg). Rectal temperatures were then measured. Each experimental group consisted of five mice. Means ± S.E. of three independent experiments are depicted. *p<0.01, ***p<0.001 compared with IgE/miR-154-5p Inh./DNP-HSA. ^#^ *p*<0.05, ^##^ *p*<0.01, ^###^ *p*<0.001 compared with IgE/Ctrl. Inh./DNP-HSA. **(B)** Immunoblot and immunoprecipitation employing lung tissue were performed. Representative blots of three independent experiments are shown. **(C)** β-hexosaminidase activity assays, histamine release assays, and qRT-PCR were performed. Serum level of PGE2 was also determined. *p<0.05; **p<0.01; ***p<0.001. Average values of three independent experiments are shown. **(D)** QRT-PCR of lung mast cell lysates was performed. ***p<0.001. Average values of three independent experiments are shown.

MiR-154-5p was necessary for vascular permeability and β-hexosaminidase activity increased by passive cutaneous anaphylaxis (PCA) (**Figure 2A**). MiR-154-5p was also necessary for hallmarks of allergic inflammation increased by PCA (**Figure 2B**) whereas an inhibitor of miR-154-5p prevented antigen from inducing interactions between FcϵRI and HDAC3 or Lyn (**Figure 2B**). Passive systemic anaphylaxis (PSA) decreased rectal temperatures in an miR-154-5p-dependent manner (**Figure 3A**). The miR-154-5p inhibitor prevented antigen from increasing hallmarks of allergic inflammation caused by PSA (**Figure 3B**). The miR-154-5p inhibitor exerted a negative effect on interactions of FcϵRIβ with HDAC3, Lyn, and SOCS1 induced by PSA (**Figure 3B**). The miR-154-5p inhibitor exerted negative effects on hallmarks of allergic inflammation increased by PSA (**Figure 3C**). Lung mast cells of PSA-induced BALB/C mice showed an increased expression of miR-154-5p (**Figure 3D**).

### MiR-154-5p Mediates Cellular Interactions

PSA can promote the tumorigenic potential of cancer cells such as B16F1 melanoma cells by enhancing cellular interactions (2, 4). We examined whether miR-154-5p could mediate cellular interactions during allergic inflammation. Culture medium from antigen-stimulated RBL2H3 cells increased hallmarks of allergic inflammation in B16F1 cells (**Figure S2A**) and lung macrophages (**Figure S2C**). It also enhanced the invasion and migration of B16F1 cells (**Figure S2B**) in an miR-154-5p-dependent manner. Immunofluorescence staining showed that the culture medium from antigen-stimulated RBL2H3 cells increased CD163 expression but decreased iNOS expression in an miR-154-5p-dependent manner in lung macrophages (**Figure S2D**). These results imply that miR-154-5p can mediate cellular interactions by regulating expression levels of soluble factors.

### MCP1, Regulated by miR-154-5p, Induces Features of Allergic Inflammation

**Figure 4:**
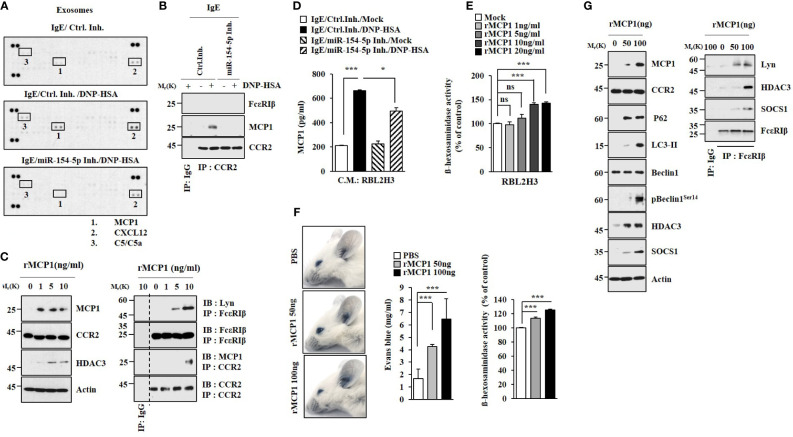
MCP1 regulated by miR-154-5p induces molecular features of anaphylaxis. **(A)**. At 24 h after transfection with the indicated inhibitor (each at 10 nM), cells were sensitized with DNP-specific IgE for 24 h followed by stimulation with DNP-HSA for 1 h. Exosomes isolated from culture medium were subjected to cytokine array analysis. **(B)** Cell lysates were subjected to immunoprecipitation. Representative blots of three independent experiments are shown. **(C)** RBL2H3 cells were treated various concentrations of mouse recombinant MCP1 protein for 1 h. Representative blots of three independent experiments are shown. **(D)** Same as **(B)** except that ELISA was performed employing culture medium. *p<0.05; ***p<0.001. Average values of three independent experiments are shown. **(E)** RBL2H3 cells were treated with various concentrations of MCP1 protein (rMCP1) for 1 h followed by β-hexosaminidase activity assays (right). ***p<0.001. Average values of three independent experiments are shown. **(F)** BALB/C moues was given an intradermal injection of rMCP1 protein at the indicated concentration along with 2% (v/v) Evans blue solution. The β-hexosaminidase activity assays employing ear tissue lysates were performed. (lower). ***p<0.001. Average values of three independent experiments are shown. **(G)** Immunoblot and immunoprecipitation were performed. Representative blots of three independent experiments are shown. NS, not significant.

Cytokine array analysis employing exosomes was performed to identify cytokines regulated by miR-154-5p in RBL2H3 cells. miR-154-5p was necessary for the increased expression of MCP1 by antigen stimulation (**Figure 4A**). Antigen stimulation also induced an interaction between CCR2, a receptor of MCP1, and MCP1 in an miR-154-5p-dependent manner in RBL2H3 cells (**Figure 4B**). Mouse recombinant MCP1 protein induced hallmarks of allergic inflammation (**Figure 4C**). ELISA of culture medium showed that antigen stimulation increased MCP1 levels in an miR-154-5p-dependent manner (**Figure 4D**). MCP1 protein (rMCP1) increased β-hexosaminidase activity in RBL2H3 cells in a dose-dependent manner (**Figure 4E**). It also increased vascular permeability and β-hexosaminidase activity in BALB/C mice (**Figure 4F**). Additionally, MCP1 protein induced molecular features associated with passive cutaneous anaphylaxis (**Figure 4G**). Antigen stimulation increased the expression of MCP1 (**Figures S3A, B**) and β-hexosaminidase activity (**Figure S3C**) in an ERK-dependent manner. N-acetyl-L-cysteine (NAC), an inhibitor of ROS formation (**Figure S3D**), exerted negative effects on expression levels of NF-kB, MCP1, and miR-154-5p increased by antigen stimulation (**Figure S3E**). MCP1 protein increased ROS levels in RBL2H3 cells (**Figure S3D**). BAY11-7085 exerted a negative effect on the expression of MCP1 increased in RBL2H3 cells by antigen (**Figure S3F**). Therefore, MCP1 may mediate cellular interactions during allergic inflammation.

### MCP1 Mediates Cellular Interactions

**Figure 5:**
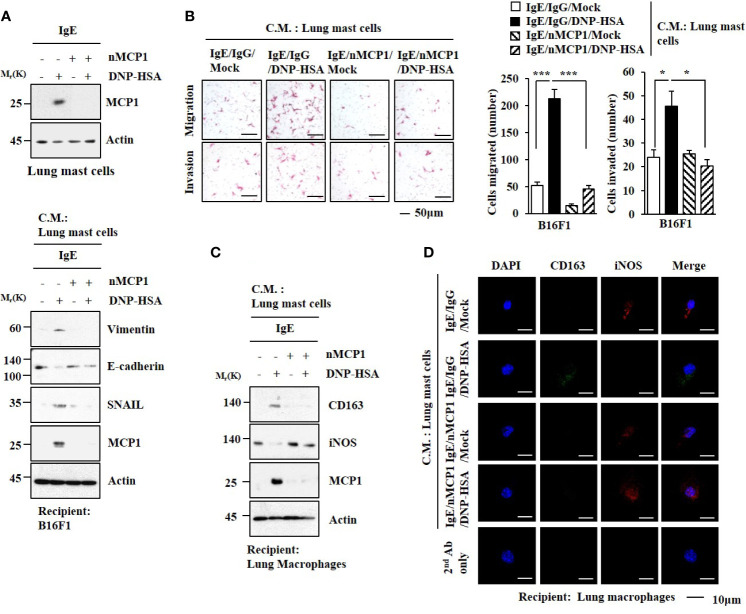
MCP1 is necessary for cellular interactions. **(A)** Lung mast cells were sensitized with DNP-specific IgE in the presence of the neutralizing MCP1 antibody (nMCP1 Ab) or isotype-matched IgG for 24 h followed by DNP-HSA stimulation for 1 h (upper). The culture medium of lung mast cells was added to B16F1cells for 24 h followed by immunoblot (lower). C.M. denotes culture medium. Representative blots of three independent experiments are shown. **(B)** Culture medium of lung mast cells was added to B16F1 cells for 48 h. Invasion and migration potentials of B16F1 cells were determined. *p<0.05; ***p<0.001. **(C)** Same as **(A)** except that culture medium was added to lung macrophages. Representative blots of three independent experiments are shown. **(D)** Immunofluorescence staining was performed.

The expression of MCP1 in lung mast cells was increased by antigen stimulation (**Figure 5A**). Culture medium from antigen-stimulated lung mast cells increased expression levels of HDAC3, MCP1, SNAIL, and Vimentin but decreased the expression of E-cadherin in B16F1 melanoma cells (**Figure 5A**). It also enhanced the invasion and migration of B16F1 cells in an MCP1-dependent manner (**Figure 5B**). Culture medium from antigen-stimulated lung mast cells increased expression levels of CD163 and MCP1 but decreased iNOS expression in macrophages (**Figures 5C, D**).

Transwell invasion analysis showed that PSA-activated lung mast cells (**Figure S4A**) and lung macrophages (**Figure S4B**) enhanced the invasion of B16F1 cells in an MCP1-dependent manner. PSA-activated lung macrophages showed increased levels of CD163 and MCP1, but a decreased level of iNOS (**Figure S4B**). Culture medium from PSA-activated lung macrophages enhanced the invasion of B16F1 cells in an MCP1-dependent manner (**Figure S4C**). MCP1 was necessary for the expression regulation of CD163 and iNOS in PSA-activated lung macrophages (**Figures S4D, E)**. Culture medium from PSA-activated lung macrophages increased the expression of SNAIL, but decreased the expression of E-cadherin in B16F1 cells in an MCP1-dependent manner (**Figure S4E**). Transwell invasion analysis showed that PSA-activated lung mast cells enhanced the invasion of lung macrophages in an MCP1-dependent manner (**Figure S4F**).

MCP1 protein (rMCP1) treatment increased CD163 expression but decreased iNOS expression in lung macrophages (**Figure S5A**). Culture medium from lung macrophages treated with MCP1 increased the expression of MCP1 and enhanced the invasion of B16F1 cells (**Figure S5A**). MCP1 protein increased β-hexosmainidase activity in lung mast cells (**Figure S5B**). Culture medium from lung mast cells treated with rMCP1 protein increased the expression levels of SNAIL and Vimentin but decreased expression of E-cadherin (**Figure S5C**). It also enhanced the invasion of B16F1cells (**Figure S5D**). Culture medium from lung mast cells treated with rMCP1 protein increased CD163 expression but decreased iNOS expression in lung macrophages (**Figures S5E, F**). These results suggest that MCP1 can mediate cellular interactions during allergic inflammation.

### MiR-154-5p and MCP1 Are Present in Exosomes

**Figure 6:**
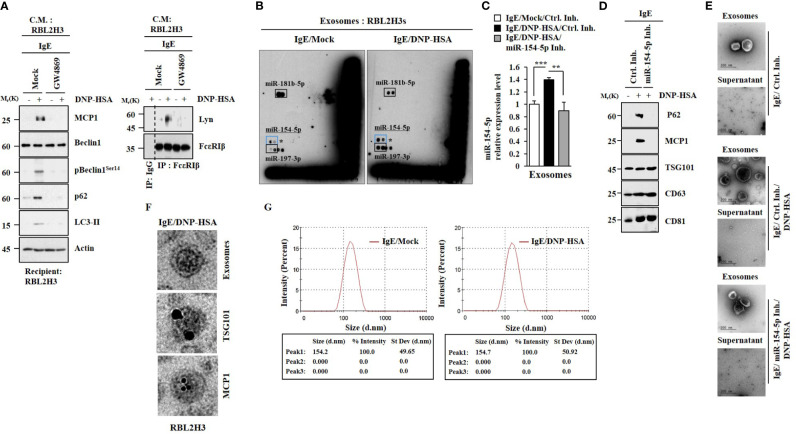
MiR-154-5p and MCP1 are present in exosomes. **(A)** IgE-sensitized RBL2H3 cells were pretreated with GW4869 (10 uM) for 24 h followed by stimulation with DNP-HSA for 1 h. The culture medium was then added to RBL2H3 cells for 24 h. Representative blots of three independent experiments are shown. **(B)** IgE-sensitized RBL2H3 cells were stimulated with DNP-HSA for 1 h. Exosomes were then isolated and subjected to miRNA array analysis. **(C)** QRT-PCR was performed. **p<0.01; ***p<0.001. Average values of three independent experiments are shown. **(D)** Immunoblot was performed. Representative blots of three independent experiments are shown. **(E)** Exosomes were seen by negative staining electron microscopy. **(F)** General appearances of isolated exosomes and immuno-gold staining images using anti-TSG101 and anti-MCP1 antibodies. Twenty-five and 10 nm gold particles indicate localizations of TSG 101 and MCP1, respectively. **(G)** Size distribution of RBL2H3-derived exosomes was determined with a Zeta sizer instrument.

GW4869, an inhibitor of exosome formation, exerted negative effects on the increased hallmarks of allergic inflammation by culture medium of antigen-stimulated RBL2H3 cells (**Figure 6A**). It also inhibited an interaction between FcϵRI and Lyn induced by culture medium of antigen-stimulated RBL2H3 cells (**Figure 6A**). Thus, exosomes may mediate cellular interactions during allergic inflammation. MicroRNA array analysis (**Figure 6B**) and qRT-PCR results (**Figure 6C**) showed the presence of miR-154-5p in exosomes of RBL2H3 cells. Immunoblot (**Figure 6D**) and Immuno-EM results (**Figure 6F**) demonstrated the presence of MCP1 in exosomes (**Figure 6F**). The presence of p62 in exosomes (**Figure 6D**) has been previously reported (4). Negative staining electron microscopic observations showed the presence of exosomes (**Figure 6E**). Sizes of exosomes were determined (**Figure 6G**). These results suggest that exosomes may mediate cellular interactions in an MCP1-dependent manner.

### Exosomes Mediate Cellular Interactions in an MCP1-Dependent Manner

**Figure 7:**
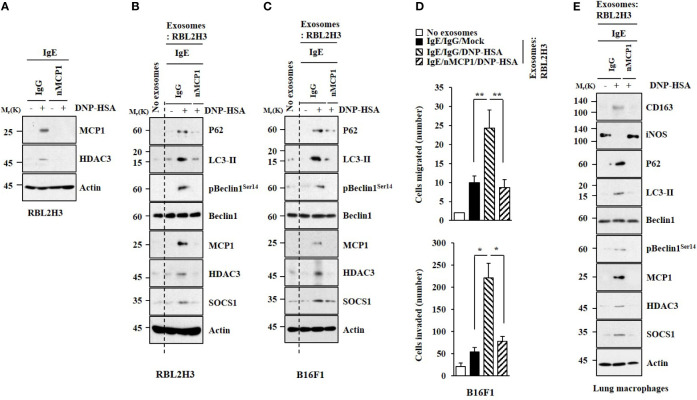
MCP1 is necessary for effects of exosomes on cellular interactions. **(A)** IgE-sensitized RBL2H3 cells were preincubated with neutralizing nMCP1 antibody (2 μg/ml) or isotype-matched IgG (2 μg/ml) for 1 h followed by stimulation with DNP-HSA for 1 h. Representative blots of three independent experiments are shown. **(B)** Same as **(A)** except that exosomes were isolated from culture medium and added to unstimulated RBL2H3 cells for 24 h. Representative blots of three independent experiments are shown. **(C)** Exosomes were added to B16F1 cells. Representative blots of three independent experiments are shown. **(D)** Migration and invasion assays were performed. *p<0.05; **p<0.01. Average values of three independent experiments are shown. **(E)** Same as **(B)** except that exosomes were added to lung macrophages. Representative blots of three independent experiments are shown.

A neutralizing MCP1 antibody exerted negative effects on expression levels of MCP1 and HDAC3 increased by antigen stimulation (**Figure 7A**). Exosomes from antigen-stimulated RBL2H3 cells increased hallmarks of allergic inflammation in unstimulated RBL2H3 cells (**Figure 7B**), B16F1 cells (**Figure 7C**), and lung macrophages (**Figure 7E**) in an MCP1-dependent manner. Exosomes from antigen-stimulated RBL2H3 cells also enhanced the invasion and migration of B16F1 cells (**Figure 7D**). We also examined whether exosomes could shuttle between cells. Unstimulated RBL2H3 cells were shown to take up PKH67-labeled exosomes (**Figure S5A**). However, fluorescence was not observed in RBL2H3 cells that took up un-labeled exosomes of unstimulated RBL2H3 cells (**Figure S6A**). Exosomes increased CD163 expression but decreased iNOS expression in lung macrophages in an MCP1-dependent manner (**Figure S6B**). Electron microscopic observation showed the insertion of exosomes into recipient cells (**Figure S6C**).

### Exosomes Induce Features of Passive Cutaneous Anaphylaxis

**Figure 8:**
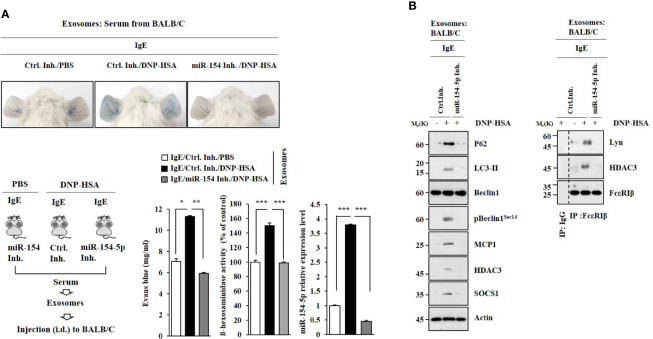
Exosomes induce features of passive cutaneous anaphylaxis. **(A)** PSA was performed as described. Exosomes were isolated from serum of BALB/C mouse of each experimental group. BALB/C mice were given an intradermal injection of exosomes (200 μg/mouse). Twenty-four hours after the injection, vascular permeability was determined. β-hexosaminidase activity assays and qRT-PCR analysis were performed. *p<0.05; **p<0.01; ***p<0.001. Average values of three independent experiments are shown. **(B)** Immunoblot and immunoprecipitation were performed. Representative blots of three independent experiments are shown.

PSA was performed as described. Exosomes were isolated from the sera of BALB/C mice in each experimental group. Exosomes enhanced vascular permeability and increased β-hexosaminidase activity in an miR-154-5p-dependent manner (**Figure 8A**). Exosomes induced molecular features of anaphylaxis in BALB/C mice in an miR-154-5p- dependent manner (**Figure 8B**). These results suggest that exosomes can mediate the effect of antigen stimulation on features of passive cutaneous anaphylaxis.

### MiR-154-5p Is Necessary for the Tumorigenic and Metastatic Potentials of Cancer Cells Enhanced by Exosomes

**Figure 9:**
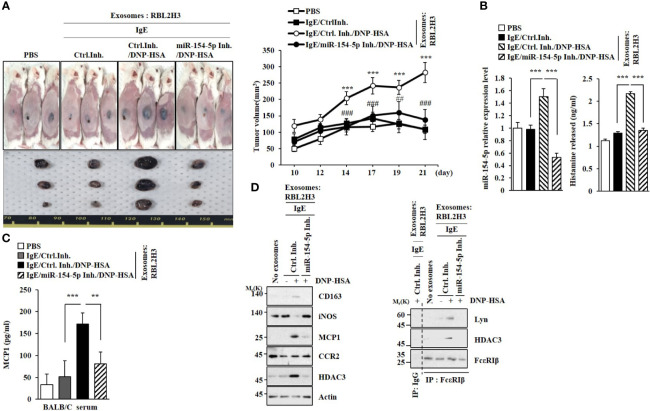
Exosomes enhance the tumorigenic potential of cancer cells in an miR-154-5p-dependent manner. **(A)** Exosomes were isolated from culture medium of RBL2H3 cells. Exosomes (50 μg) were mixed with B16F1 cells (2X10^5^). BALB/C mice were given a subcutaneous injection. ***p<0.001 compared with IgE/Ctrl. Inh. ^##^ *p*<0.01, ^###^ *p*<0.001 compared with IgE/Ctrl. Inh./DNP-HSA. **(B)** QRT-PCR analysis was performed. The level of histamine released was also determined. ***p<0.001. Average values of three independent experiments are shown. **(C)** Serum level of MCP1 was determined. **p<0.01; ***p<0.001. Average values of three independent experiments are shown. **(D)** Immunoblot and immunoprecipitation were performed. Representative blots of three independent experiments are shown.

Exosomes from antigen-stimulated RBL2H3 cells enhanced the tumorigenic potential of B16F1 melanoma cells in an miR-154-5p-dependent manner (**Figure 9A**). The enhanced tumorigenic potential by exosomes was accompanied by increased levels of histamine released (**Figure 9B**) and MCP1 expression (**Figure 9C**). The enhanced tumorigenic potential was accompanied by increased expression of CD163 and decreased expression of iNOS in an miR-154-5p-dependent manner (**Figure 9D**). Exosomes from antigen-stimulated RBL2H3 cells induced molecular features of allergic inflammation (**Figure 9D**, right). Exosomes from antigen-stimulated RBL2H3 cells also enhanced the metastatic potential of B16F1 melanoma cells in an miR-154-5p-dependent manner (**Figure S7A**). Additionally, those exosomes induced molecular features of allergic inflammation in an miR-154-5p-dependent manner (**Figure S7B**). These increases of MCP1 level (**Figure S7C**), histamine released (**Figure S7D**), and β-hexosaminidase activity (**Figure S7D**) might have contributed to the enhanced metastatic potential of B16F1 melanoma cells.

### Reactive Oxygen Species Mediate Cellular Interactions During Allergic Inflammation

Exosomes of antigen-stimulated RBL2H3 cells promoted tumorigenic (**Figure 9**) and metastatic potentials (**Figure S7**) of mouse melanoma cells in an miR-154-5p-dependent manner. Antigen stimulation increased ROS levels (**Figure S3D**) and MCP1 expression (**Figure S3E**), suggesting that ROS might mediate cellular interactions. GW4869 prevented antigen stimulation from increasing ROS levels in RBL2H3 cells (**Figure S8A**). NAC prevented antigen from increasing ROS levels in lung mast cells (**Figure S8B**). NAC exerted negative effects on the expression of MCP1 (**Figure S8C**) and β-hexosaminidase activity (**Figure S8D**) increased by antigen in lung mast cells. ROS production was necessary for increased expressions of MCP1, SNAIL, and Vimentin (**Figure S8E**) and invasion and migration potentials of B16F1 cells (**Figure S8F**) by culture medium of antigen-stimulated lung mast cells. NAC also prevented the culture medium of antigen-stimulated lung mast cells from regulating expression levels of MCP1, CD163 and iNOS in lung macrophages (**Figures S8G, H**). Thus, ROS could mediate cellular interactions by regulating the expression of MCP1.

## Discussion

miRNAs have been shown to play critical roles in allergic inflammation (23, 24). The role of p62, a selective receptor of autophagy, in allergic inflammation has been reported (4). miRNA analysis was performed to identify targets of p62. miR-31-5p and miR-154-5p were also regulated by p62 (**Figure 1A**). miR-154-5p mediated PCA (**Figure 2**) and PSA (**Figure 3**). Roles of miR-154-5p in cellular proliferation (25), differentiation (26), and cardiac remodeling (27) have been previously reported. The level of miR-31 has been found to be higher in lung tissues of asthmatic mice than in controls (28). miR-31-5p is expressed in airway epithelia and it recruits neutrophils during allergic airway inflammation (29). It is probable that miR-31-5p can mediate anaphylaxis. miR-342-3p was found to be negatively regulated by p62 (**Figure 1A**). These data imply that miR-342-3p might act as a negative regulator of allergic inflammation.

Passive systemic anaphylaxis can promote tumorigenic and metastatic potentials (19, 30). This implies an interaction between cancer cells and mast cells. Conditioned medium from LLC (Lewis lung carcinoma) cells can increase Runx2/VEGF/Dusp5 expressions in mast cells, and promote tumor angiogenesis (31). The conditioned medium of antigen-stimulated lung mast cells can enhance migration and invasion potentials of B16F1 melanoma cells in an SOCS1-depedent manner (2). SOCS1 regulates anaphylactic shock viscera injury processes (32). Additionally, asthmatic bronchial epithelium shows an increased expression of SOCS1 (33). Thus, it is reasonable that the interaction between cancer cells and immune cells may regulate tumorigenic potential of cancer cells.

Cytokine array analysis revealed that miR-154-5p was necessary for the expression of MCP1 increased by antigen stimulation (**Figure 4A**). ROS production contributes to an increased expression of MCP1 (34). OVA-induced allergic airway inflammation is also closely associated with the increased expression of MCP1 (35). The HDAC3-MCP1 axis regulates allergic skin inflammation (36). Allergic asthma induced by house dust mite allergen can lead to an increased expression of MCP1 (37). In our study, we found that ROS-MAPK signaling regulated expression level of MCP1 (**Figure S2**). MCP1 protein stimulation also induced features of allergic reactions *in vitro* (**Figure 4C**) and passive cutaneous anaphylaxis (**Figures 4F, G**) in an IgE-independent manner. MCP1 may induce features of passive systemic anaphylaxis such as rectal temperature decrease. It would be necessary to identify targets of recombinant MCP1 protein. TargetScan predicted miR-124-5p, miR-196-5p, and miR27a-3p as negative regulators of MCP1, suggesting that these miRNAs might inhibit allergic inflammation.

**Figure S3D** clearly shows that antigen stimulation could increase the production of ROS (**Figure S3D**). Additionally, we demonstrated that ROS/MAPK/NF-kB signaling regulated the expression of MCP1 and miR-154-5p (**Figure S3**). ROS signaling is necessary for mast cell activation (38). NF-kB/MAPK signaling can mediate allergic inflammation promoted by mast cell activation (39). The effect of ROS/MAPK/NF-kB signaling on allergic inflammation merits further study. Induction of autophagy by rapamycin can lead to an increased expression of MCP1 in inflamed tissues (40). Thus, inhibition of autophagy by CQ or 3-MA might inhibit anaphylaxis by decreasing the expression of MCP1.

Experiments employing culture medium showed that miR-154-5p (**Figure S2**) and MCP1 (**Figure 5**) were necessary for cellular interactions involving cancer cells, mast cells and macrophages. We showed the presence of miR-154-5p (**Figure 6B**) and MCP1 (**Figures 6D, F**) in the exosomes of RBL2H3 cells. Since ROS signaling was necessary for the increased expression of MCP1, ROS signaling might mediate cellular interactions.

A previous study has shown that exosomes contribute to the pathogenesis of asthma (11). Additionally, exosomes from the broncho alveolar lavage fluid (BALF) of sarcoidosis patients can induce the expression of CCL2 (MCP1) (41). Here, we showed that exosomes from antigen-stimulated RBL2H3 cells increased the expression of MCP1 in B16F1 melanoma cells (**Figure 7C**) and lung macrophages (**Figure 7E**). Mast cell-derived exosomes can induce mesenchymal transition in airway epithelial cells (42). Culture medium from PSA-activated lung mast cells (**Figure 5A**) and lung macrophages (**Figure S4E**) increased the expression of SNAIL but decreased the expression of iNOS in B16F1 cells in an MCP1-dependent manner. Since ROS signaling regulates the expression of MCP1, future research is warranted to examine effects of exosomes on ROS signaling.

MiR-154-5p was necessary for tumorigenic (**Figure 9A**) and metastatic potentials (**Figure S6A**) of B16F1 melanoma cells enhanced by exosomes isolated from RBL2H3 cells. Downstream targets of miR-154-5p could act as regulators of allergic inflammation both *in vitro* and *in vivo*. Exosomal miRNAs are involved in cellular communications (43). Identification of more exosomal miRNAs and proteins is necessary to develop a better understanding of anaphylaxis mediated by cellular interactions.

MiRNA array analysis showed that miR-342 was a potential target of miR-154-5p. An miR-342 mimic negatively regulated passive cutaneous anaphylaxis and passive systemic anaphylaxis (personal observations). Several reports have suggested a role of miR-342 as a tumor suppressor (44, 45). It will be necessary to examine the presence of miR-342 in exosomes and identify downstream targets of miR-342-3p.

In this study, we showed novel roles of miR-154-5p and MCP1 in cellular interactions during allergic inflammation.

## Data Availability Statement

The original contributions presented in the study are included in the article/**Supplementary Material**. Further inquiries can be directed to the corresponding author.

## Ethics Statement

The animal study was reviewed and approved by Institutional Animal Care and Use Committee (IACUC) of Kangwon National University.

## Author Contributions

YmK, HSJ, and DJ designed the study. MK, HJ, YjK, and MJ performed the experiments. DJ wrote the manuscript. All authors contributed to the article and approved the submitted version.

## Funding

This work was supported by National Research Foundation Grants (2017M3A9G7072417, 2018R1D1A1B07043498, 2020R1A2C1006996, and 2020R1A6AA1306651011), a grant from the BK21 plus four Program.

## Conflict of Interest

The authors declare that the research was conducted in the absence of any commercial or financial relationships that could be construed as a potential conflict of interest.
