# Supplementary material for: MiR-154-5p-MCP1 Axis Regulates Allergic Inflammation by Mediating Cellular Interactions
Source: Front Immunol. 2021 May 31;12:663726. doi: 10.3389/fimmu.2021.663726 (PMC8201518; doi:10.3389/fimmu.2021.663726)
Supplement: Supplementary file 1 [file DataSheet_1.pdf]

Supplement to

MiR-154-5p-MCP1 axis regulates allergic inflammation by mediating cellular interactions

Misun Kim[1], Hyein Jo[1], Yoojung Kwon[1], Myeong Seon Jeong[1,2], Hyun Suk Jung[1], Youngmi Kim[3], Dooil Jeoung[1]

SUPPLYMENTAL METHODS

β-hexosaminidase Activity Assays.

RBL2H3 cells were grown on 2% (w/v) BSA-coated 96 well plates (2×10^5 cells/well) and sensitized for 16 h with DNP-specific IgE (100 ng/ml). The culture medium was replaced with Tyrodes' assay buffer (119 mM NaCl, 4.74 mM KCl, 2.5 mM CaCl2, 1.19 mM MgSO4, 10 mM 4-2-hydroxyethyl-1-piperazineethane sulfonic acid (HEPES), 5 mM glucose, 0.1% (w/v) BSA, pH7.3). IgE-sensitized cells were then preincubated with the above buffer for 15 min. The cells were then stimulated with DNP-HSA (100 ng/ml) for 1 h. The supernatant (80 μl) was incubated with an equal volume of substrate solution (1 mM p-nitrophenyl N-acetyl-beta-D-glucosamine in 0.05 M citrate buffer, pH 4.5) for 1 h at 37 °C. The enzyme reaction was stopped by the addition of 0.05 M sodium bicarbonate buffer (pH 10.0) and the reaction product was measured at 450 nm. To determine the total amount of β-hexosaminidase released, the remaining cells were lysed by assay buffer containing 1% (v/v) Triton X-100 prior to incubation with substrate. For β-hexosaminidase assay using tissue lysates, tissue was frozen in liquid nitrogen to preserve protein structure and homogenized using lysis buffer (62.5 mM Tris-HCl, pH 6.8, 2% (w/v) SDS, 10% (v/v) glycerol, 50 mM dithiothreitol, 0.01% (w/v) bromophenol blue, 10 mM NaF, 1% (v/v) protease inhibitor mixture (Roche), 1 mM sodium orthovanadate). Vortexing and centrifugation at 10,000 X g for 15 min at 4°C were followed. Supernatant was then obtained and used as tissue lysates. Eighty μl of tissue lysates (50 μg/ml) were incubated with an equal volume of substrate solution for 1 h at 37 °C. The enzyme reaction was stopped by the addition of 0.05 M sodium bicarbonate buffer (pH 10.0) and the reaction product was measured at 450 nm.

Immunoblot and Immunoprecipitation.

Cells were solubilized by lysis buffer (50mM Tris-HCl, 150mM NaCl, 1% NP40, pH 8.0, 10 mM NaF, 1% (v/v) protease inhibitor mixture (Roche), 1 mM sodium orthovanadate). For immunoblot, lysates were mixed with 2 X sample buffer, boiled for 5 min, and denatured proteins (10-20 μg) were analyzed on 10% SDS-PAGE. After electrophoresis, proteins were transferred to PVDF membrane. After transfer, membrane was incubated with blocking

solution (5% BSA in TBS-T buffer) for 30 minutes to block non-specific binding. The membrane was then incubated with primary antibody (diluted in 2% BSA) at 4°C for overnight, followed by washing three times with TBS-T for 15 min and incubation with anti-mouse or anti-rabbit horseradish peroxidase-conjugated antibody for 1 h. Immunodetection was performed using an enhanced chemiluminescent substrate solution (GenDEPOT, USA).
To isolate tissue lysates, tissue was frozen in liquid nitrogen to preserve protein structure and homogenized using lysis buffer. Vortexing and centrifugation at 10,000 X g for 15 min at 4ºC were followed. Supernatant was then obtained and used as tissue lysates.
For immunoprecipitations, cell lysates or tissue lysate (100-200 μg) were immunoprecipitated with respective primary antibody (0.2 -2 μg) for overnight at 4℃. Twenty μl of Protein A/G PLUS-Agarose (Santa Cruz) was then added and incubation was continued for 1 hour at 4℃. Beads were washed three times with lysis buffer, 2X sample buffer was added. Samples were then denatured (100 °C for 5min) and analyzed 10% SDS-PAGE, followed by immunoblot.

Figure S1

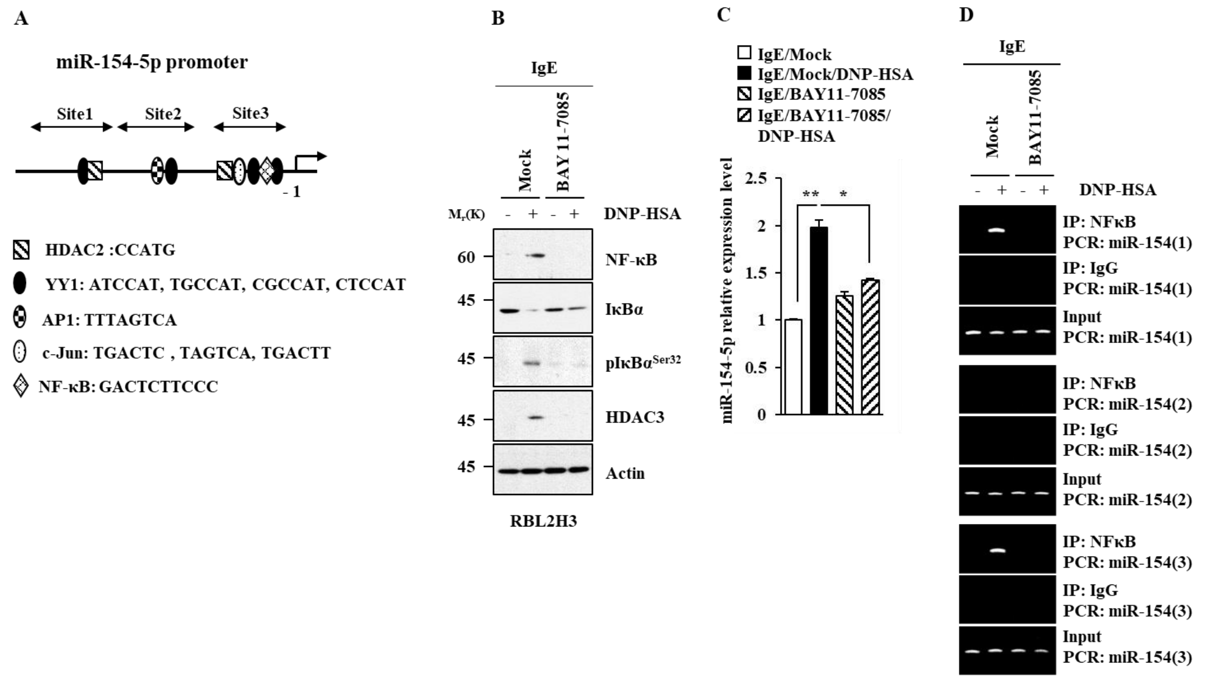

FIGURE S1. NF-κB is necessary for the increased expression of miR-154-5p by antigen stimulation. (A) Shows potential binding sites of transcriptional factors in the promoter sequences of miR-154-5p. IgE-sensitized RBL2H3 cells were pretreated with BAY11-7085 (20 μM) for 1 h. Cells were then stimulated with DNP-HSA for 1 h. Immunoblot (B), QRT-PCR analysis (C), and ChIP assay (D) were performed. *, $p<0.05$; **, $p<0.01$. Numbers in parentheses denote primer binding sites.

Figure S2

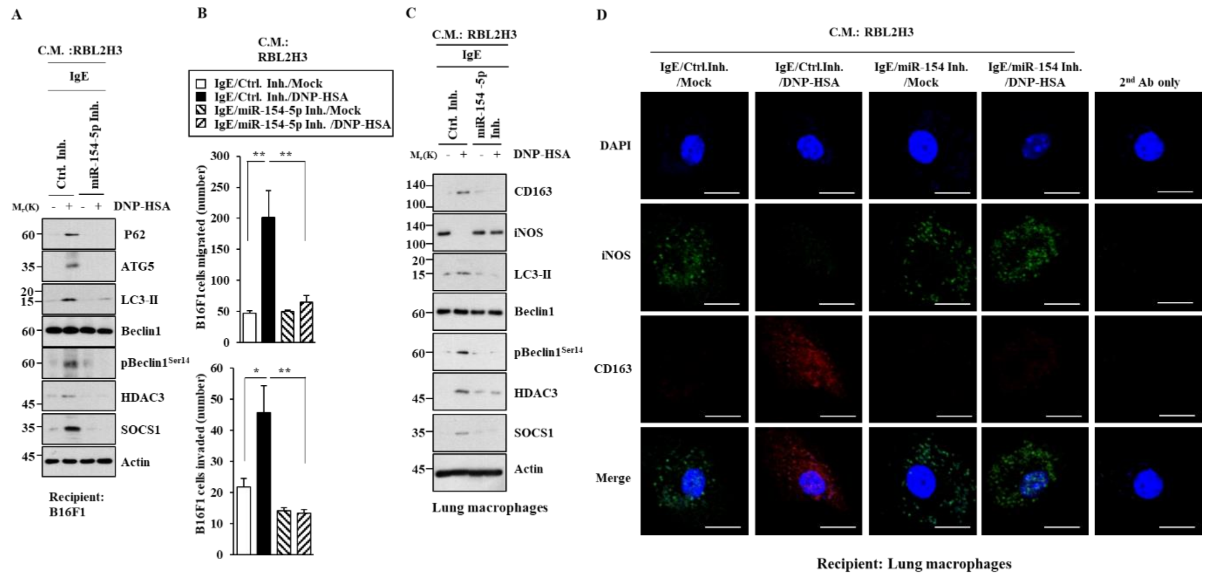

FIGURE S2. MiR-154-5p is necessary for cellular interactions. **(**A) At 24 h after transfection with the indicated inhibitor (each at 10 nM), cells were sensitized with DNP-specific IgE for 24 h followed by stimulation with DNP-HSA for 1 h. Culture medium was then added to B16F1 cells and incubated for 8 h. Representative blots of three independent experiments are shown. (B) At 48 h after addition of culture medium to B16F1 cells, invasion and migration potentials were determined. *, *p*<0.05; **, *p*<0.01. Average values of three independent experiments are shown. (C) Same as (A) except that culture medium was added to lung macrophages. (D) Immunofluorescence staining was performed.

Figure S3

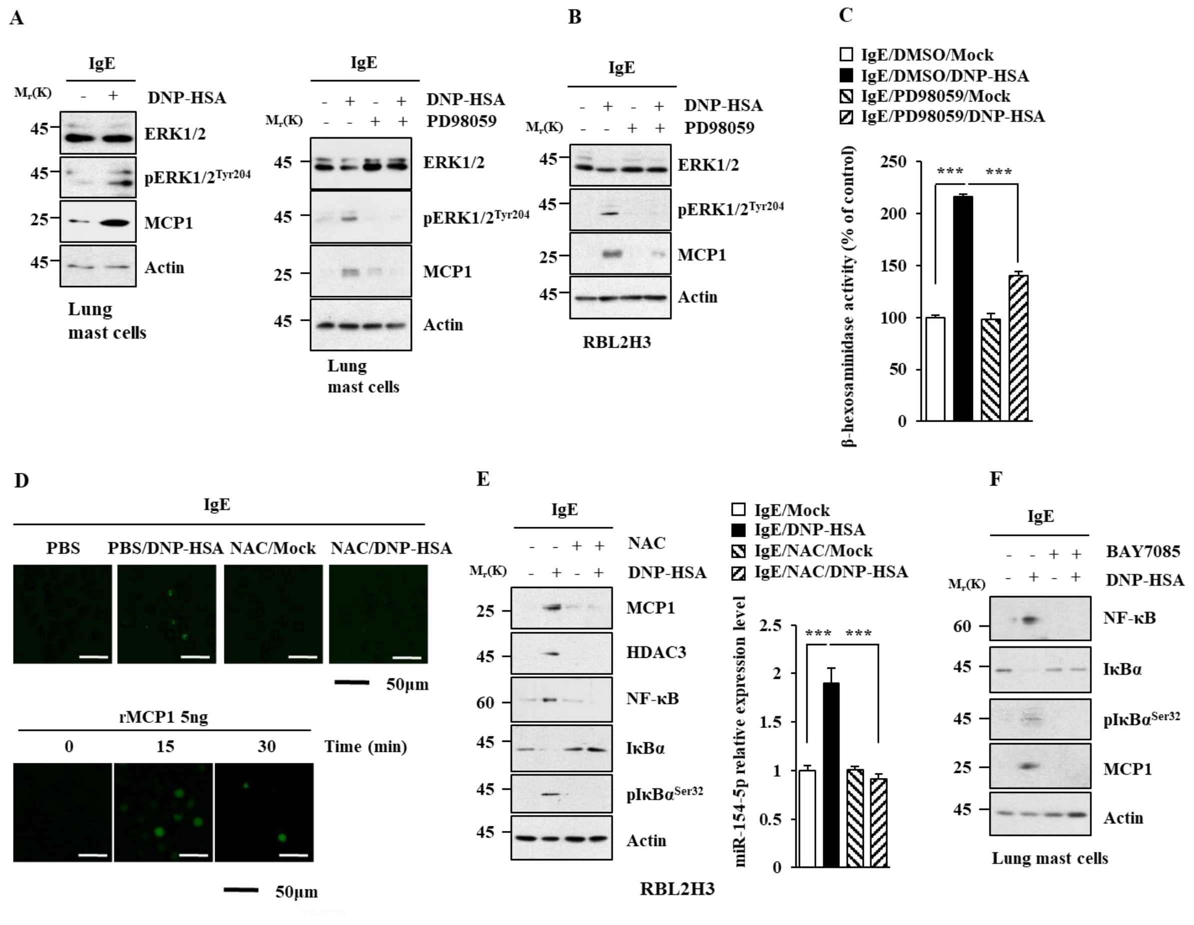

FIGURE S3. ROS-MAPK-NF-kB signaling regulates MCP1 expression. (A) IgE-sensitized lung mast cells were stimulated with DNP-HSA for 1 h (left). IgE-sensitized lung mast cells were pretreated with PD98059 (20 μM) for 1 h followed by DNP-HSA stimulation for 1 h (right). Representative blots of three independent experiments are shown. (B) Same as (A) except that RBL2H3 cells were employed. (C) The β-hexosaminidase activity assays were performed. ***, *p*<0.001. Average values of three independent experiments are shown. (D) IgE-sensitized RBL2H3 cells were pretreated with NAC (500 μM) for 2 h followed by DNP-HSA stimulation for 1 h. DCFH-DA (5 μM) was added at 30 minutes after addition of DNP-HSA (upper). RBL2H3 cells were treated with rMCP1 protein (5 ng) for various time intervals (lower). DCFH-DA was added along with MCP1 protein (lower). (E) Immunoblot and qRT-PCR analysis were performed. ***, *p*<0.001. (F) IgE-sensitized RBL2H3 cells were pretreated with BAY11-7085 (20 μM) for 1 h followed by stimulation with DNP-HSA for 1 h. Representative blots of three independent experiments are shown.

Figure S4

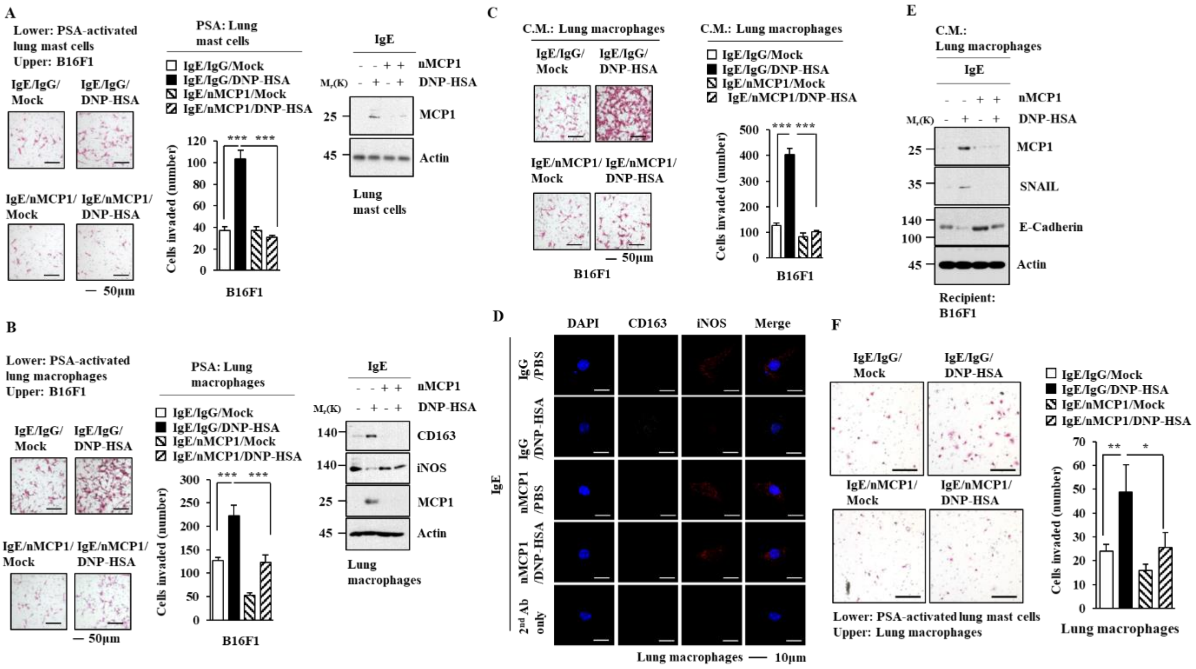

FIGURE S4. PSA-activated lung mast cells and lung macrophages enhance the invasion of B16F 1 melanoma cells. Transwell was employed to examine the effect of MCP1 on cellular interactions. Upper well contains B16F1 cells. Lower well contains lung mast cells (A) or lung macrophages (B). Invasion potential of B16F1 cells was determined. ***, p<0.001. Immunoblot was performed employing lysates of PSA-activated lung mast cells (A) or lung macrophages (B). (C) Lower well contains culture medium of PSA-activated lung macrophages. Invasion potential of B16F1 cells was determined. ***, p<0.001. (D) Immunofluorescence staining of PSA-activated lung macrophages was performed. (E) PSA-activated lung macrophages were isolated from BALB/C mouse of each experimental group. The culture medium of lung macrophages was added to B16F1 cells for 24 h followed by immunoblot. (F) Lower well contains PSA-activated lung mast cells. Upper well contains lung macrophages. Invasion potential of lung macrophages was determined. *, p<0.05; **, p<0.01. Representative figures of three independent experiments are shown. Average values of three independent experiments are shown.

Figure S5

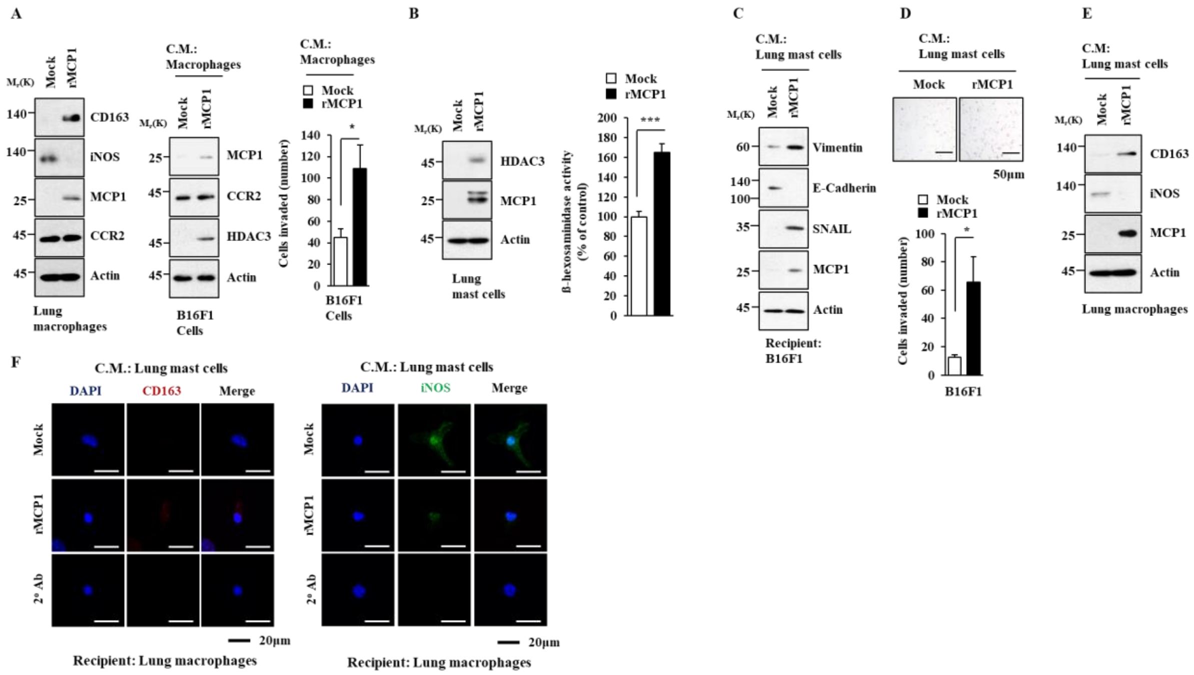

FIGURE S5. Recombinant MCP1 protein mediates cellular interactions. (A) Lung macrophages were treated with rMCP1 protein (10 ng/ml) for 2 h followed by immunoblot (left). Culture medium of lung macrophages treated with rMCP1 proteins was added to B16F1 cells for 24 h (middle). Invasion potential of B16F1 cells was determined (right). *, $p<0.05$. Representative blots of three independent experiments are shown. (B) Lung mast cells were treated with rMCP1 protein. Immunoblot (left) and β-hexosaminidase activity assay (right) were performed. Average values of three independent experiments are shown. (C) Culture medium of lung mast cells treated with rMCP1 proteins for 2 h was added to B16F1 cells for 24 h. Representative blots of three independent experiments are shown. (D) Same as (C) except that invasion potential of B16F1 cells was determined. Average values of three independent experiments are shown. (E) Same as (B) except that culture medium was added to lung macrophages for 24 h. (F) Same as (E) except that immunofluorescence staining was performed.

Figure S6

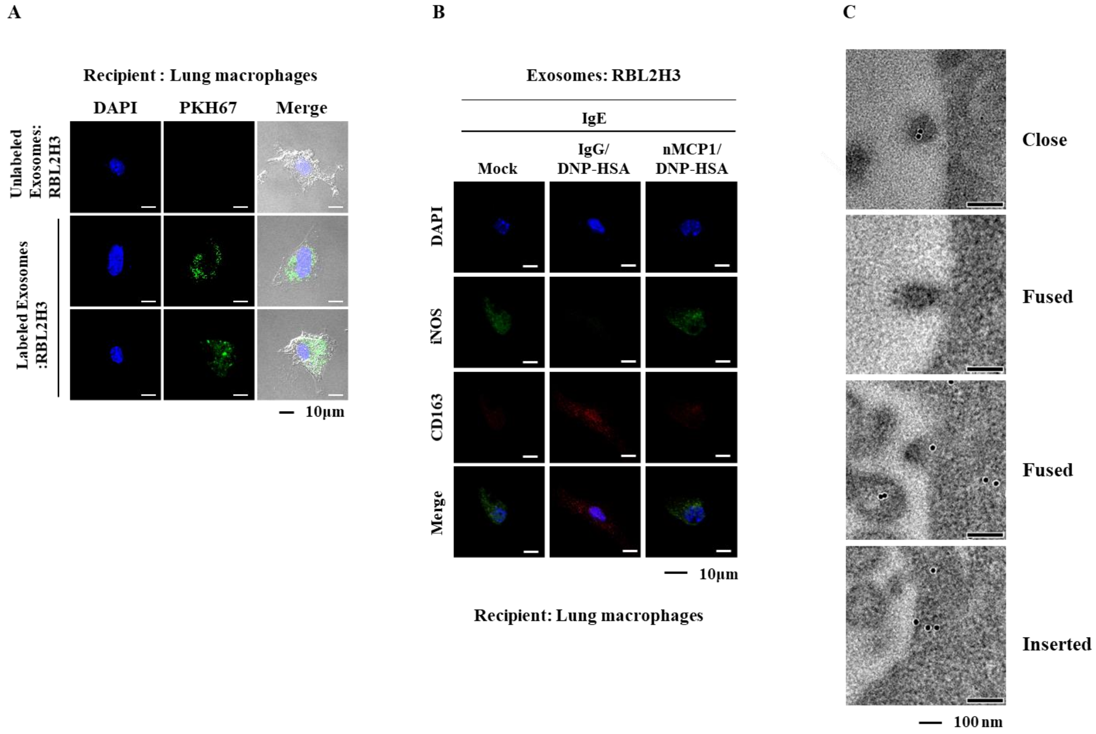

FIGURE S6. Exosomes shuttle between cells, and MCP1 is necessary for effects of exosomes on hallmarks of M2 macrophages polarization. (A) Exosomes isolated from antigen-stimulated RBL2H3 cells were labeled with PKH67 dye (green fluorescence). PKH67-labeled exosomes were added to lung macrophages. Cells were then visualized using a confocal laser scanning microscope (LSM710). (B) Exosomes from antigen-stimulated RBL2H3 cells preincubated with neutralizing MCP1 antibody or isotype-matched IgG were added to lung macrophages for 24 h. (C) Immunogold-staining EM shows localization of CD63 in the lumen of the exosome near the membrane of a target cell (upper panel), outside of exosomes near the cell (middle panel), and its potential location within cells (bottom panel).

Figure S7

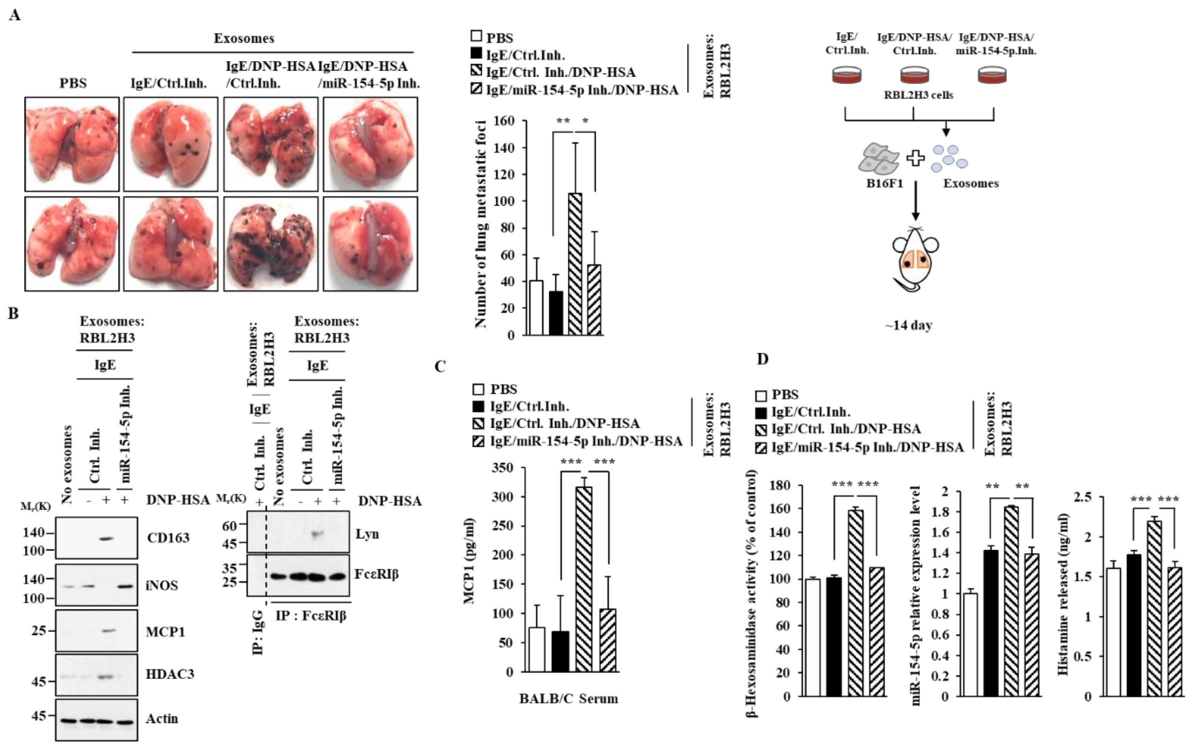

FIGURE S7. Exosomes enhance the metastatic potential of cancer cells in an miR-154-5p-dependent manner. (A) Exosomes (50 µg) isolated from culture medium of RBL2H3 cells were mixed with B16F1 cells (2X10^5). BALB/C mice were given an intravenous injection. *, p<0.05; **, p<0.01. (B) Immunoblot and immunoprecipitation were performed. Representative blots of three independent experiments are shown. (C) Serum level of MCP1 was determined. ***, p<0.001. Average values of three independent experiments are shown. (D) MiR-154-5p expression, the amount of histamine released, and β-hexosaminidase activity were determined. **, p<0.01; ***, p<0.001. Average values of three independent experiments are shown.

Figure S8

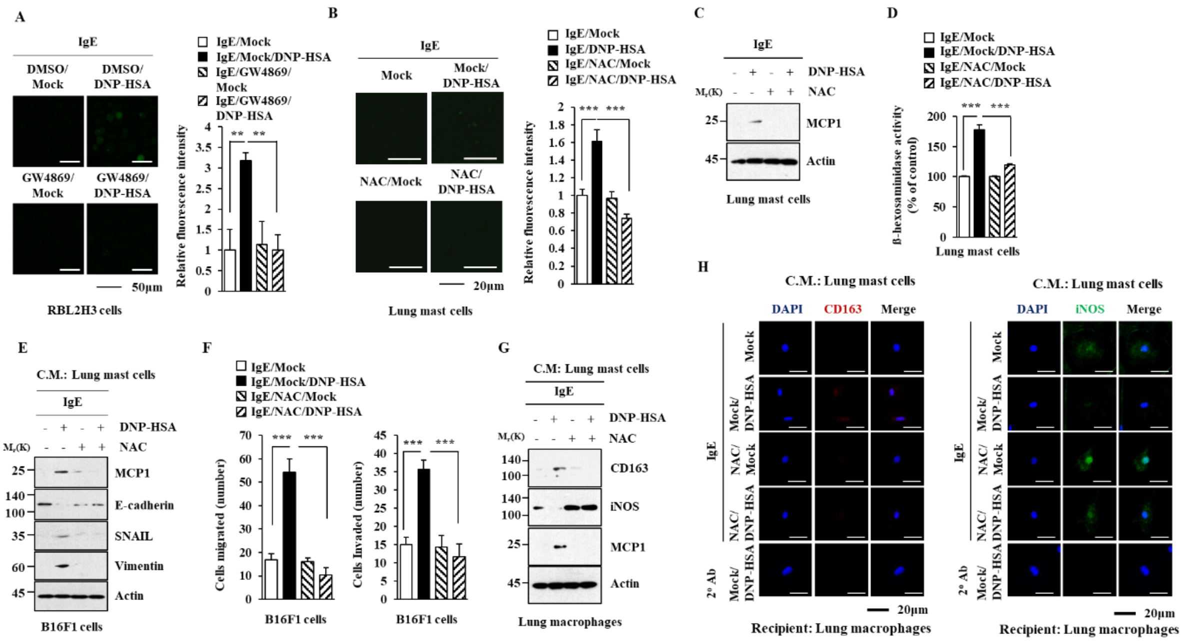

FIGURE S8. Reactive oxygen species (ROS) regulate cellular interactions. (A) IgE-sensitized RBL2H3 cells were pretreated with GW4869 (10 μM) for 24 h followed by stimulation with DNP-HSA for 1 h. DCFH-DA (5 μM) was added at 30 minutes after the addition of DNP-HSA. **, *p*<0.01. Representative figures of three independent experiments are shown. (B) Same as (A) except that cells were pretreated with NAC (500 μM). ***, *p*<0.001. (C) Immunoblot was performed. Representative blots of three independent experiments are shown. (D) β-hexosaminidase activity assays were performed. Average values of three independent experiments are shown. (E) Culture medium of lung mast cells was added to B16F1 cells for 24 h. Representative blots of three independent experiments are shown. (F) Invasion and migration potentials of B16F1 cells were determined. ***, *p*<0.001. Average values of three independent experiments are shown. (G) Same as (E) except that culture medium of lung mast cells was added to lung macrophages for 24 h. Representative blots of three independent experiments are shown. (H) Same as (G) except that immunofluorescence staining was performed.
